# Integrated characterization of filler tobacco leaves: HS–SPME–GC–MS, E-nose, and microbiome analysis across different origins

**DOI:** 10.1186/s40643-024-00728-w

**Published:** 2024-01-18

**Authors:** Mingzhu Zhang, Dongfeng Guo, Haiqing Wang, Guanglong Wu, Naihong Ding, Yaqi Shi, Jinlong Zhou, Eryong Zhao, Xingjiang Li

**Affiliations:** 1 Key Laboratory for Agricultural Products Processing, School of Food and Biological Engineering, Hefei University of Technology, Danxia Road 485#, Hefei City, 230601 Anhui Province China; 2 China Tobacco Anhui Industrial Co., Ltd., Huangshan Road 606#, Hefei City, 230088 Anhui Province China

**Keywords:** Filler tobacco leaves, Volatile organic compounds, Microbial community, Chemical constituents, Yunnan region

## Abstract

This study delves into the aroma characteristics and microbial composition of filler tobacco leaves (FTLs) sourced from six distinct cigar-growing regions within Yunnan, China, following standardized fermentation. An integrated approach using gas chromatography-mass spectrometry (GC–MS), electronic nose (E-nose), and microbiome analysis was employed for comprehensive profiling. Results derived from Linear Discriminant Analysis (LDA) using E-nose data confirmed the presence of notable variability in flavor substance profiles among the FTLs from six regions. Additionally, GC–MS was used to discern disparities in volatile organic compound (VOC) distribution across FTLs from these regions, identifying 92, 81, 79, 58, 69, and 92 VOCs within each respective sample set. Significantly, 24 VOCs emerged as pivotal determinants contributing to the heterogeneity of flavor profiles among FTLs from diverse origins, as indicated by Variable Importance for the Projection (VIP) analysis. Furthermore, distinctions in free amino acid content and chemical constituents were observed across FTLs. Of noteworthy significance, solanone, isophorone, durene, (-)-alpha-terpineol, and 2,3'-bipyridine exhibited the strongest correlations with microbiome data, with fungal microorganisms exerting a more pronounced influence on metabolites, as elucidated through two-way orthogonal partial least-squares (O2PLS) modeling. These findings provide a theoretical and technical basis for accurately evaluating the synchronization of FTLs in aromas and fermentation processes, and they will enhance the quality of fermented FTLs and foster the growth of the domestic cigar tobacco industry ultimately.

**Graphical Abstract:**

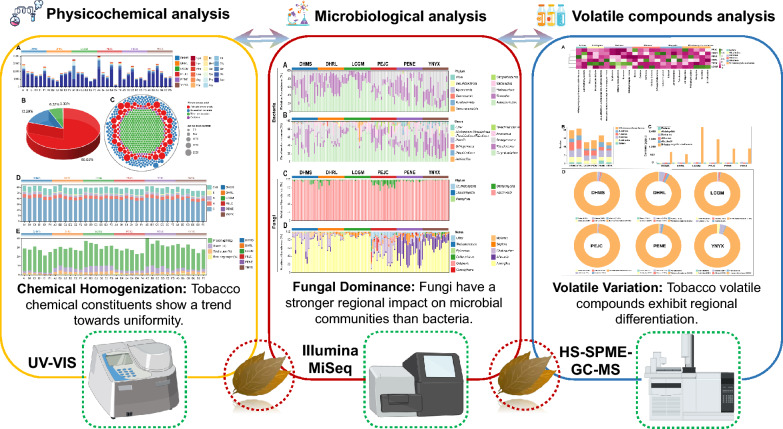

**Supplementary Information:**

The online version contains supplementary material available at 10.1186/s40643-024-00728-w.

## Introduction

A cigar, revered both domestically and internationally, is a unique tobacco product crafted from tobacco leaves employed as the filler, binder, and wrapper. Cigars are celebrated for their robust kick, rich and full-bodied aroma, and a taste spectrum that ranges from fragrant sweetness to bitter undertones. The history of cigar usage can be traced back to the indigenous peoples of the Americas. Following Columbus's discovery of the New World, cigars found their way to various corners of the globe through Europe (Tso 1972). A traditional hand-rolled cigar comprises three integral components: the filler, nestled at the core of the cigar, the binder situated in the middle, and the wrapper outermost. Notably, the filler tobacco, occupying the central position within the cigar, takes precedence as the primary component. It usually makes up 70 to 85 percent of the total weight of a cigar and has a crucial influence on the style and character of the cigar (Chen et al. 2023). Furthermore, the composition and quantity of aroma-producing constituents within the filler tobacco leaves exhibit variations across different regions of production, constituting a significant determinant of divergent style attributes (Wang et al. 2014b). In addition to these chemical factors, the microbial community structure of tobacco leaves plays a crucial role in determining the quality and character of cigars. Microbial communities, consisting of a variety of bacteria and fungi, actively participate in the fermentation process, influencing the development of aroma compounds and the overall sensory profile of tobacco (Ning et al. 2023a, b; Zhang et al. 2023a). The diversity and dynamics of these microbial populations are closely linked to environmental factors such as climate, soil composition, and agricultural practices, varying in different tobacco-growing regions.

As consumer standards in China continue to rise, a rapidly expanding market for cigars, particularly in the middle- to high-end segments, has taken shape. Currently, major regions for cigar tobacco leaf production abroad are concentrated in areas such as the Caribbean, Brazil, the United States, and Indonesia (Li et al. 2019). Cigars, composed entirely of tobacco leaves, exhibit a greater dependence on raw materials compared to traditional cigarettes. However, due to their relatively recent cultivation history and limited research, domestically produced cigar tobacco leaves still fall short of the quality standards achieved in renowned producing regions abroad. This quality gap poses a significant hurdle to the development of China's cigar industry. The cultivation of cigar tobacco leaves demands specific conditions related to temperature, rainfall, soil quality, and cultivation techniques. Only a handful of regions worldwide possess the capability to produce high-quality tobacco leaves. Countries like Cuba, the Dominican Republic, and Ecuador, all characterized by tropical rainforest or tropical monsoon climates, serve as prime examples. Currently, China's primary cigar-producing regions are primarily located in Hainan, Yunnan, Sichuan, Hubei, and other areas. Various factors, including tobacco variety (Wang et al. 2020), cultivation practices, curing methods (Liu et al. 2021b), and fermentation processes, have been identified as influencing the quality of tobacco raw materials used in cigar tobacco leaf production. During fermentation, the microbial community structure undergoes significant changes, which are essential for the development of the tobacco's distinct aroma and flavor. The microbial activity during this stage is responsible for the breakdown of precursor substances into aromatic compounds, thus playing a pivotal role in defining the unique characteristics of cigar tobacco leaves. Proper fermentation techniques can significantly enhance the visual appeal, physical attributes, chemical composition, and sensory allure of tobacco leaves. Fermentation plays a crucial role in ensuring the uniformity of production and enhancing the overall quality of cigar tobacco leaves. It effectively mitigates certain quality imperfections present in the original tobacco, intensifies the tobacco's aroma, and substantially elevates the smoking experience (Niu et al. 2020). After a reasonable fermentation process, the accessibility and sensory flavor of tobacco leaves will be significantly improved.

To address the complex nature of cigar tobacco leaves, especially in terms of their microbial communities and flavor compounds, analytical techniques play a crucial role. The electronic nose (E-nose), designed to simulate human olfactory perception, is widely utilized across various industries, including food, beverages, environmental monitoring, and medical diagnostics, due to its capability to rapidly and non-destructively gauge the aroma characteristics of different tobacco products. Although the E-nose provides expedient aroma profiling, it falls short in offering detailed structural information about compounds and generally has lower sensitivity and specificity. In contrast, headspace solid-phase microextraction gas chromatography–mass spectrometry (HS–SPME–GC–MS) excels in the detailed analysis and quantification of both volatile and semi-volatile compounds, playing a crucial role in highlighting the chemical variations of tobacco leaves from different regions. However, this approach can be both time-consuming and intricate. Additionally, high-throughput sequencing, used for swiftly and accurately identifying microbial community structures, including bacteria and fungi, is particularly significant in tobacco leaf research. This method allows for an exploration of microbial diversity and functionality, providing insights into how these microorganisms affect flavor formation and fermentation processes.

The tobacco leaf itself and the fermentation process are the main sources of the fragrance compounds present in cigar tobacco leaves. Analyzing and evaluating the aroma components of cigar tobacco leaves can lay a crucial foundation for both production and quality assurance. The raw materials used to make cigar tobacco leaves and the features of the fermentation process utilized can both be directly understood through these scent components (Rong et al. 2021). Tobacco leaves house a multitude of aromatic compounds, albeit with relatively low concentrations. These compounds include fragrance compounds and fragrance precursor chemicals (Liu et al. 2022). Throughout the phases of tobacco leaf growth, development, modulation, and aging, these fragrance precursors undergo conversion and degradation, ultimately forming aroma compounds that directly influence the tobacco's flavor. Ketones, for instance, represent vital aromatic molecules formed through the degradation of carotenoids (Li et al. 2021). Depending on the aromatic functional groups they contain, these compounds can be categorized into a number of classes, including ketones, aldehydes, acids, esters, alcohols, and alkanes. In the realm of tobacco, terpenoids, such as siberanoids, play a significant role. The oxidative breakdown of the carbon chain of siberanoid molecules results in the direct production of aldehydes and ketones such as solanone, geranylacetone, and solanedione.

However, in the current stage of research in China, the focus in the cigar domain primarily centers on areas like tobacco leaf preparation (Zhang et al. 2020b) and fermentation methods (Zhang et al. 2021). There exists a limited body of work comparing the differences in chemical composition and microbial community structure of tobacco leaves following fermentation in various growing regions. To explore inherent quality disparities and levels of homogeneity among filler tobacco leaves (FTLs) from six distinct regions in Yunnan, this study undertook a comparative analysis and clustering analysis encompassing main chemical components, aroma compounds, and microbiome information. Collectively, these technologies, each with its unique strengths and limitations, work in tandem to provide a comprehensive understanding of the microbiological and chemical properties of cigar tobacco leaves. This integrated study is to offer technical support for the development and research direction of domestically produced high-quality cigar tobacco leaves.

## Materials and methods

### Sample preparation

The samples used in this study were generously provided by the China Tobacco Anhui Industry Co., Ltd. The study analyzed fermented tobacco leaf samples (FTLs) sourced from six geographically distinct areas within Yunnan Province, China. These areas include: DHMS (Mang City, Dehong Prefecture), DHRL (Ruili City, Dehong Prefecture), LCGM (Dai-Va Autonomous County of Gengma, Lincang City), PEJC (Hani-Yi Autonomous County of Jiangcheng, Pu'er City), PENE (Hani-Yi Autonomous County of Ninger, Pu'er City), and YNYX (Yuxi City). Each region is characterized by its unique environmental and cultural attributes, contributing to the diverse profiles of the tobacco leaves. All the samples selected for this study had undergone a thorough fermentation process. From each region, six different grades of FTLs were carefully selected, as detailed in Additional file 1: Table S1. To ensure a representative sampling approach, five locations were used to collect FTLs from the high, middle, and bottom layers of the fermentation heap, as shown in Additional file 1: Fig. S1. Samples obtained from these three layers were combined to create a single biological replicate, aiming to encompass the full range of variation in the fermentation process. Subsequently, all collected samples were rapidly frozen and subjected to grinding in liquid nitrogen. These processed samples were then stored under two different temperature conditions: −20 °C and −80 °C. This preparation was carried out to facilitate physical–chemical analysis and DNA extraction in subsequent stages of the study.

### Determination of free amino acids and chemical constituents

We followed a modified version of the method by Gao et al. (Gao et al. 2021) to quantify the free amino acids (FAAs) content. Briefly, 0.4 g of finely chopped and thoroughly mixed samples were homogenized in 8 mL of sulfosalicylic acid (4%, w/v) and centrifuged at 3 °C for 10 min at 12,000 g. The resulting supernatant was filtered through a 0.22 µm membrane filter after undergoing another round of centrifugation with the same condition. Subsequently, an automatic amino acid analyzer (S-433D, Sykam, Eresing, Germany) was employed to analyze the filtered supernatant for FAA determination.

In accordance with the Chinese National Standard GB/T 5009.5-2016, we conducted an assessment of the protein content. Specifically, we employed a Kjeldahl apparatus (Hanon Technologies CO., Ltd. Shandong, China) for protein content determination. Total sugar and reducing sugar contents were assessed using ultraviolet spectroscopy (Shanghai Metash Instruments CO., Ltd. Shanghai, China). Starch content was determined through an iodine colorimetry method. Additionally, we utilized an Elemental Analyzer (Flash Smart, Thermo Fisher, America) to detect the chemical elements, including carbon (C), hydrogen (H), sulfur (S), and nitrogen (N).

### Analysis of HS–SPME–GC–MS and E-nose

The HS–SPME–GC–MS analysis procedure closely followed the method outlined by Liu et al. (Liu et al. 2020), with minor improvements incorporated. Initially, 0.5 g of the sample was mixed with 1 μL of phenylmethyl acetate in 20 mL vials. Subsequently, the mixture underwent a series of steps: equilibration (70 ℃, 20 min), extraction (70 ℃, 35 min), and desorption (250 ℃, 3 min) within the Agilent 8890 GC and Agilent 7000D gas chromatography-tandem mass spectrometry system. This system was equipped with an HP-5MS column (30 m × 0.32 mm i.d. and 0.25 µm film thickness; J&W Scientific, CA, USA). Experimental conditions using the HP-5MS column included a flow rate of 0.8 mL/min, helium as the carrier gas (99.99% purity), and an initial column temperature of 60 ℃, maintained for 2 min. Subsequently, the temperature was raised to 180 ℃ at a rate of 3 ℃/min and held at 180 ℃ for an additional 2 min. The temperature was further increased to 260 ℃ at a rate of 6 ℃/min, with a final hold at 260 ℃ for 2 min. Using the following equations, the volatile organic compound content (*C*_*v*_*,* μg/μL) was determined:

1 $$C_v \, = \,\frac{S_v }{{S_a }}\, \times \,C_a$$

where the peak areas of the volatile organic compound and phenylmethyl acetate, are denoted by *S*_*v*_, *S*_*a*_, respectively. And *C*_*a*_ (μg/μL) is the content of phenylmethyl acetate.

To assess the total odor difference among the FTLs samples, we made slight modifications to the method originally described by Li et al. (2023) Specifically, 0.5 g of diced and uniformly mixed samples were placed in a 20 mL headspace vial. These vials were preheated for 10 min at 60 °C and then analyzed using an electronic nose (PEN3, Airsense Analytics GmbH, Schwerin, Germany). The PEN3 system incorporates 10 metal oxide gas sensors, each designed to detect specific components, such as W1C (aromatic components), W5S (nitrogen oxides), W3C (aromatic components), W6S (hydrogen), W5C (alkanes and aromatic components), W1S (short-chain alkanes), W1W (inorganic sulfides), W2S (alcohols, aldehydes, ketones, etc.), W2W (aromatic components and organic sulfides), and W3S (long-chain alkane). 400 mL/min of injection flow rate and 150 s of analysis duration were the test parameter configurations.

### DNA extraction, polymerase chain reaction, and bioinformatics analysis

The HiPure Soil DNA Kit (Magen, Guangzhou, China) was employed for the isolation of total DNA following the manufacturer's guidelines. Amplification of ITS sequences was executed using primers ITS1F (5′-CTTGGTCATTTAGAGGAAGTAA-3′) and ITS2 (5′-GCTGCGTTCTTCATCGATGC-3′) (Toju et al. 2012). For the amplification of bacterial 16S rRNA genes, we utilized the universal bacterial primers 515F (5′-GTGYCAGCMGCCGCGGTAA-3′) and 806R (5′- GGACTACNVGGGTWTCTAAT-3′) (Apprill et al. 2015; Parada et al. 2016). To distinguish between individual samples, these primers were equipped with PacBio barcode sequences. The PCR procedure was conducted in triplicate using a 20 μL reaction mixture in the MyCycler Thermal Cycler (Bio-Rad, USA), following the protocol detailed in reference (Liu et al. 2023). Subsequently, a DNA library was constructed with the SMRTbell TM Template Prep Kit (PacBio, Menlo Park, CA, USA) by combining the products in an equimolar ratio. GenDenovo Honour Biotechnology Co., Ltd. (Guangzhou, China) sequenced the purified SMRTbell libraries on the PacBio Sequel platform. After the initial sequencing data underwent filtration using FASTP software (version 0.18.0) (Guo et al. 2017), we merged the original sequencing data using FLASH software (version 1.2.11) (Magoč and Salzberg 2011). Clustering for the identification of operational taxonomic units (OTUs) was performed using UPARSE software (version 9.2.64) with a 97% similarity threshold (Edgar 2013). We set the comparison threshold at 80% and assigned taxonomy to the OTUs using the Silva database for bacteria and the UNITE database for fungi. To ensure data accuracy, we identified and eliminated chimeric sequences using the UCHIME method (Edgar et al. 2011). Finally, QIIME software (version 1.9.1) was employed for the evaluation of diversity and the generation of species abundance tables across various classification levels (Caporaso et al. 2010).

### Data analysis

The data were examined using analysis of variance (ANOVA) to assess the significance of the difference (*p* < 0.05) using SPSS 20.0 (version R27.0.1.0), which was utilized for statistical analysis. Linear Discriminant Analysis (LDA) was performed using WinMuster software (version 1.6.2.23) and Variable Importance for the Projection (VIP) analysis was performed using SIMCA software (version 14.1). The Pearson correlation coefficient and two-way orthogonal partial least-squares (O2PLS) were used to analyze the relationship between volatile taste compounds and microorganism genus, and a dynamic real-time interactive online platform, ChiPlot (https://chiplot.online/), was used to make relevant heat map. The link between considerably different flavor compounds and microbial genera was examined using the Pearson correlation coefficient. Gephi software (version 0.10) was used for visualization.

## Results

### Analysis of chemical constituents for different origins filler tobacco leaves

**Fig. 1:**
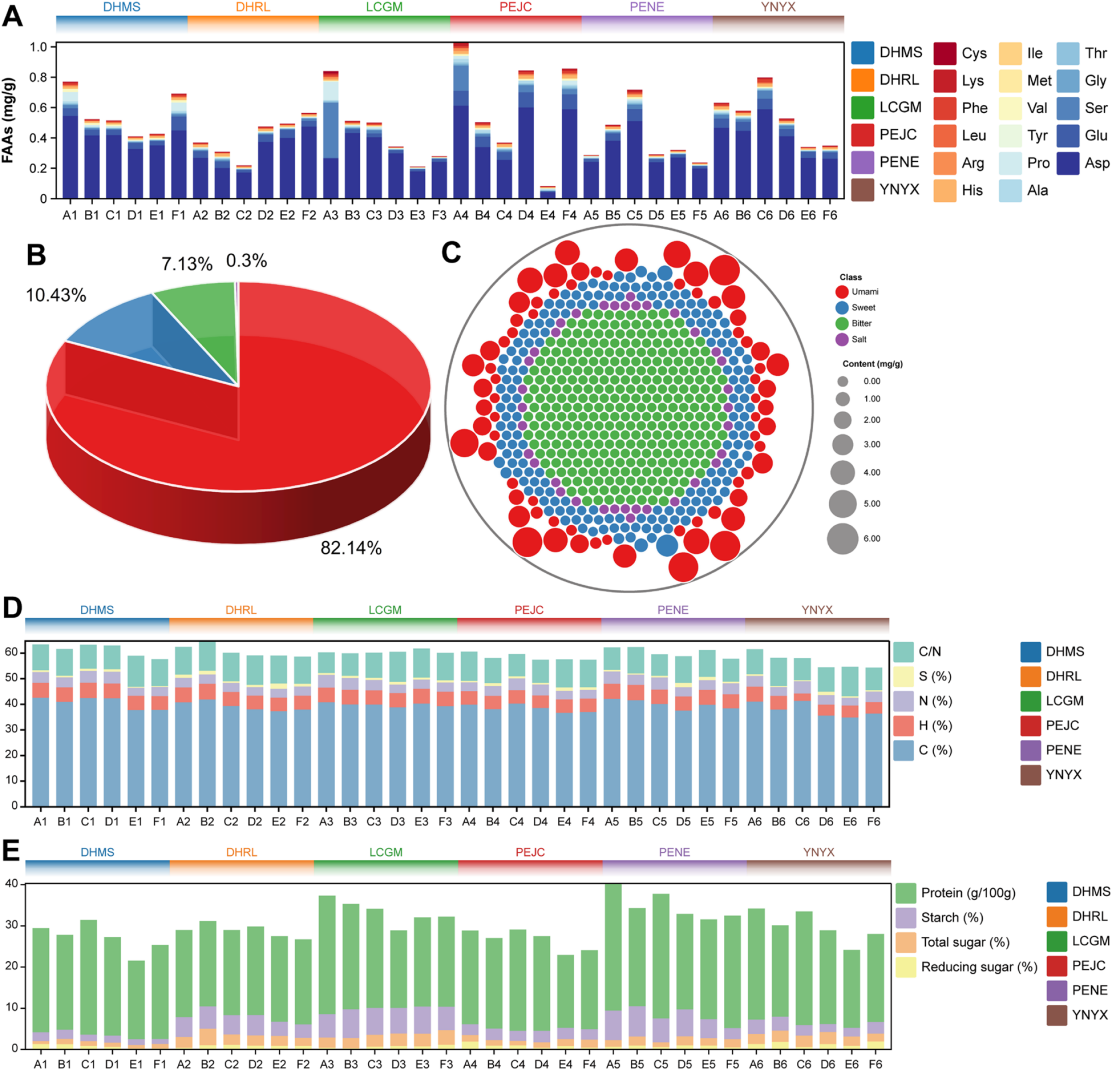
Analysis of the free amino acids and organic acids of the filler tobacco leaves. **A** Free amino acids concentrations. **B** Proportion, species, and amount distribution **C** of different classes of free amino acids. **D** Chemical elements proportion. **E** Difference of chemical constituents in each group of filler tobacco leaves. (See data Additional file 1: Tables S2, S3, S4 and S5). DHMS, DHRL, LCGM, PEJC, PENE, and YNYX, respectively, denote distinct geographical region names, The cigar tobacco samples are categorized into six distinct grades, designated as A (Fi-B-1-Bt-S), B (Fi-B-2-Bt-S), C (Fi-C-1-Bt-L), D (Fi-C-2-Bt-L), E (Fi-X-3-Bt-M), and F (Fi-X-4-Bt-M). Subscripted numbers 1 through 6, which denote their geographical origins: 1 for DHMS, 2 for DHRL, 3 for LCGM, 4 for PEJC, 5 for PENE, and 6 for YNYX, respectively

FAAs, serving as pivotal precursors for aroma compounds, make significant contributions to both the sensory experience and nutritional characteristics of the final product (Shen et al. 2011). In this study, we conducted a comprehensive analysis of FAAs across six distinct groups of FTLs originating from different regions. As illustrated in Fig. 1A, there were notable variations in the levels of FAAs observed among these six FTL groups. Notably, the PEJC group exhibited the highest cumulative FAAs content, while the PENE group displayed the lowest, marking a substantial disparity of 13.44 mg/g between them. FAAs can be systematically categorized into four primary groups: umami amino acids, encompassing Aspartic (Asp) and Glutamic (Glu); sweet amino acids, which include Serine (Ser), Glycine (Gly), Threonine (Thr), Alanine (Ala), and Proline (Pro); bitter amino acids, encompassing Tyrosine (Tyr), Valine (Val), Methionine (Met), Isoleucine (Ile), Histidine (His), Arginine (Arg), Leucine (Leu), Phenylalanine (Phe), and Lysine (Lys); and salt amino acid (Cysteine, Cys) (Wang et al. 2014a). Our analysis revealed that these categories contribute significantly to the overall FAAs content in FTLs. Specifically, umami amino acids constituted the majority, accounting for 82.14% of the total FAAs. This was followed by sweet amino acids at 10.43%, bitter amino acids at 7.13%, and salt amino acids, which were the least prevalent, comprising only 0.30% of the total FAAs content. This distribution underscores the dominance of umami amino acids in FTLs, highlighting their critical role in the flavor profile of the product (Fig. 1B). It is particularly noteworthy that Aspartic (Asp) emerged as the most abundant among the FAAs, exhibiting an average concentration exceeding, 3.00 mg/g.

To gain a visual understanding of how different types of FAAs contribute to the overall taste profile of FTLs, we constructed a circular stacked plot depicting FAAs with diverse flavor attributes (Fig. 1C). Notably, Asp and Glu, while present in smaller quantities among the various amino acid types within FTLs, exhibited the highest concentrations, potentially imparting an umami taste to these leaves. Conversely, sweet and bitter amino acids showcased a broader spectrum of amino acid types but were characterized by relatively lower concentrations. In this study, a majority of bitter and sweet amino acids displayed similar concentrations, a phenomenon likely attributed to microbial fermentation, which results in a more balanced flavor profile within cigar tobacco leaves. Furthermore, we conducted a comprehensive analysis of four chemical elements and four chemical constituents within FTLs sourced from six distinct regions. Principal Component Analysis (PCA) was subsequently performed based on the proportions of these chemical elements and chemical constituents, with the PCA score plot presented in Additional file 1: Fig. S2. Remarkably, the three principal components collectively accounted for 71.80% of the total variance. Specifically, PC1 elucidated 37.40% of the overall variance, while PC2 contributed 21.20% to the variance among samples, and PC3 explained 13.2% of the sample variance. The PCA effectively discerned and illuminated the variations in FTLs derived from six diverse regions. The PCA results, corroborated by subsequent statistical analyses, revealed a pronounced trend towards chemical uniformity in terms of elemental composition and constituents among the FTLs. Upon comprehensive examination, it was observed that in all samples from DHMS, DHRL, LCGM, PEJC, PENE, and YNYX, the C content was consistently high, yet without significant disparities (*p* > 0.05). Similarly, the N content exhibited no notable differences (*p* > 0.05), leading to a uniform C/N ratio across the cigar tobacco samples from these regions. This uniformity implies a certain level of homogenization in the flavor profile, aroma, and combustion characteristics of Yunnan’s cigar tobaccos, as shown in Fig. 1D. Furthermore, the concentrations of reducing sugars and proteins also displayed homogenized patterns. During fermentation, reducing sugars are metabolized by microorganisms into acids and other compounds, enhancing the flavor of the tobacco leaves. Proteins, on the other hand, are broken down into smaller amino acids and compounds, affecting the aroma and flavor of the cigar tobacco. This observation lends further support to the earlier inference (Fig. 1E). Nonetheless, it is imperative to emphasize the significant variations in the levels of H, S, total sugar, and starch, as delineated in Additional file 1: Tables S4, and S5.

### Analysis of volatile organic compounds

In contrast to traditional sensory evaluation methods, electronic sensor technology offers distinct advantages, including simplicity, speed, and efficient analysis, in objectively assessing the olfactory and taste characteristics of samples (Geană et al. 2020). Consequently, E-noses were employed in this study to investigate the sensory properties of FTLs. The results obtained from the LDA, as depicted in Additional file 1: Fig. S3, revealed that the response values of the ten E-nose sensors exhibited a degree of similarity among DHMS, DHRL, LCGM, PEJC, PENE, and YNYX, implying comparable olfactory characteristics within the six groups of FTLs. However, it is essential to note that this degree of similarity did not reach a high level of homogeneity. To delve deeper into the aroma profile of FTLs, we employed HS–SPME–GC–MS for the analysis of VOCs present in FTLs. As delineated in Additional file 1: Fig. S4A, a total of 170 compounds were identified, encompassing 11 esters, 9 aldehydes, 37 ketones, 57 alkenes, 20 alcohols, 22 N-heterocyclic carbenes, 3 phenols, and 11 other compounds across the six groups of FTLs. Variations in the composition of VOCs can substantially contribute to the diverse flavor characteristics observed in FTLs. The types and concentrations of VOCs exhibited significant variability among the six groups of FTLs (Additional file 1: Fig. S4B, C, and D). Notably, DHMS and YNYX have the same number of VOCs (92). Nevertheless, the total concentration of volatiles in DHMS was the lowest (1134.58 μg/μL), whereas YNYX exhibited the highest total concentration (4190.99 μg/μL). Conversely, PEJC featured the fewest types (58) of VOCs, albeit with a moderate total concentration (2172.44 μg/μL) (Additional file 1: Table S6). In terms of concentration, n-heterocyclic carbenes emerged as the most abundant VOCs in each sample group, despite their relatively lower diversity compared to ketones and alkenes.

**Fig. 2:**
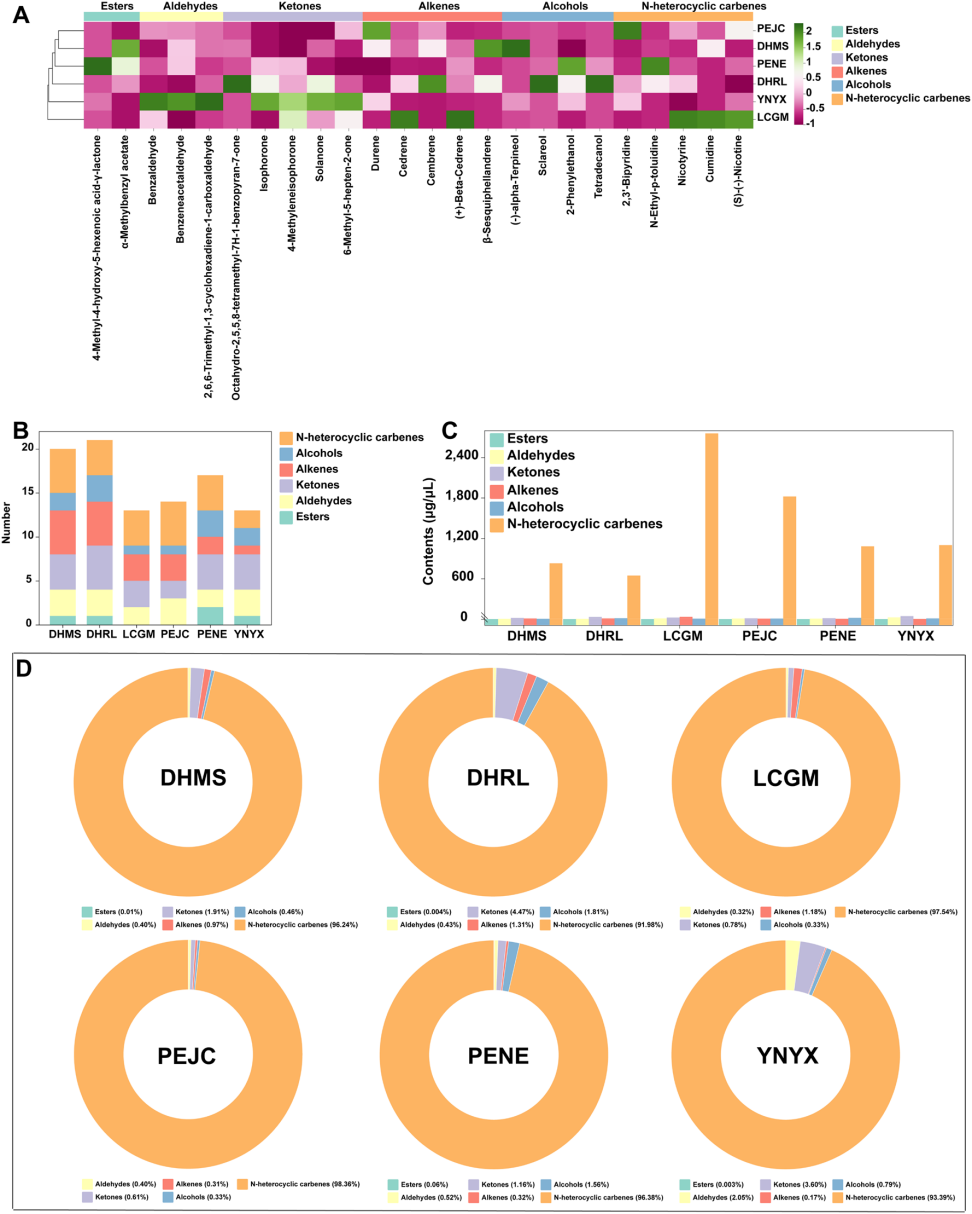
Analysis of volatile organic compounds (VIP > 1.0) of the filler tobacco leaves identified and analyzed by HS–SPME–GC–MS and PLS-DA. **A** Volatile organic compound concentrations and clustering results. **B** Types of volatile organic compounds. **C** Concentrations of each group of volatile organic compounds. **D** Proportion of each group of volatile organic compounds

Furthermore, Partial Least Squares-Discrimination Analysis (PLS-DA) was conducted based on the VOCs data, yielding R2X, R2Y, and Q2 values of 0.689, 0.761, and 0.561, respectively, indicating the robustness of the PLS-DA model. To assess the model's quality, permutation tests (*n* = 200) were performed, resulting in R2Y = 0.529 and Q2Y = −0.386, affirming the model's goodness of fit. Considering a threshold VIP > 1, we identified 24 VOCs as markers (Additional file 1: Table S7). These included two esters (4-methyl-4-hydroxy-5-hexenoic acid-*γ*-lactone, *α*-methylbenzyl acetate), three aldehydes (benzaldehyde, benzeneacetaldehyde, 2,6,6-trimethyl-1,3-cyclohexadiene-1-carboxaldehyde), five ketones (octahydro-2,5,5,8-tetramethyl-7H-1-benzopyran-7-one, isophorone, 4-methyleneisophorone, solanone, 6-methyl-5-hepten-2-one), five alkenes (durene, cedrene, cembrene, ( +)-beta-cedrene, *β*-Sesquiphellandrene), four alcohols ((−)-alpha-terpineol, sclareol, 2-phenylethanol, tetradecanol), and five n-heterocyclic carbenes (2,3'-bipyridine, n-ethyl-p-toluidine, nicotyrine, cumidine, (s)-(-)-nicotine) (Fig. 2A). These 24 selected substances exhibited varying levels across the six groups of FTLs. Notably, DHRL featured the highest number of volatile compound types (VIP > 1, 21 types) but had the lowest total concentration (700.60 μg/μL). Conversely, LCGM shared the same types of volatile compounds as YNYX (VIP > 1, 13 types), yet LCGM exhibited the highest total volatile compound concentration (2825.21 μg/μL) (Fig. 2B, C). Concerning the content of volatile organic compounds, n-heterocyclic carbenes dominated, constituting over 90% of the content in each group, primarily composed of (s)-(-)-nicotine (Fig. 2D). This finding aligns with previous research results (Tso 1972). Nicotine, a crucial factor influencing tobacco leaf quality, can heighten the harshness and spiciness of the leaf when its content is excessively high. During the fermentation process, microorganisms contribute to the degradation of a portion of nicotine in tobacco leaves, enhancing the overall smoking experience. Simultaneously, these microorganisms can also break down other undesirable components in tobacco leaves, such as nitrosamines, tar, and proteins.

### Diversity and differences in microbial community composition

To assess the microbial biogeography associated with FTLs, we conducted a comprehensive examination of microbial taxonomic profiles derived from an extensive dataset comprising 108 FTLs fermentation samples. Our objective was to discern distinct microbial characteristics that may be linked to the diverse geographic origins of these FTLs. Our analysis unveiled a diverse microbial landscape within FTLs from various regions. In the case of DHMS samples, we identified 103 bacterial amplicon sequence variants (OTUs) and 144 fungal OTUs. Conversely, DHRL samples exhibited 78 bacterial OTUs and 122 fungal OTUs. Meanwhile, the LCGM category showcased 84 bacterial OTUs and 154 fungal OTUs, indicating its unique microbial composition. In stark contrast, PEJC displayed 92 bacterial OTUs and an impressive 190 fungal OTUs, signifying a rich and distinct microbial presence. PENE, another region of interest, exhibited 106 bacterial OTUs and 158 fungal OTUs. Finally, YNYX featured 102 bacterial OTUs and 107 fungal OTUs. In all, our careful examination resulted in the annotation of a total of 103 bacterial genera and 132 fungal genera in the FTLs fermentation ecosystem, identifying them at the level of genus classification (for detailed information, please refer to Additional file 1: Table S8).

**Fig. 3:**
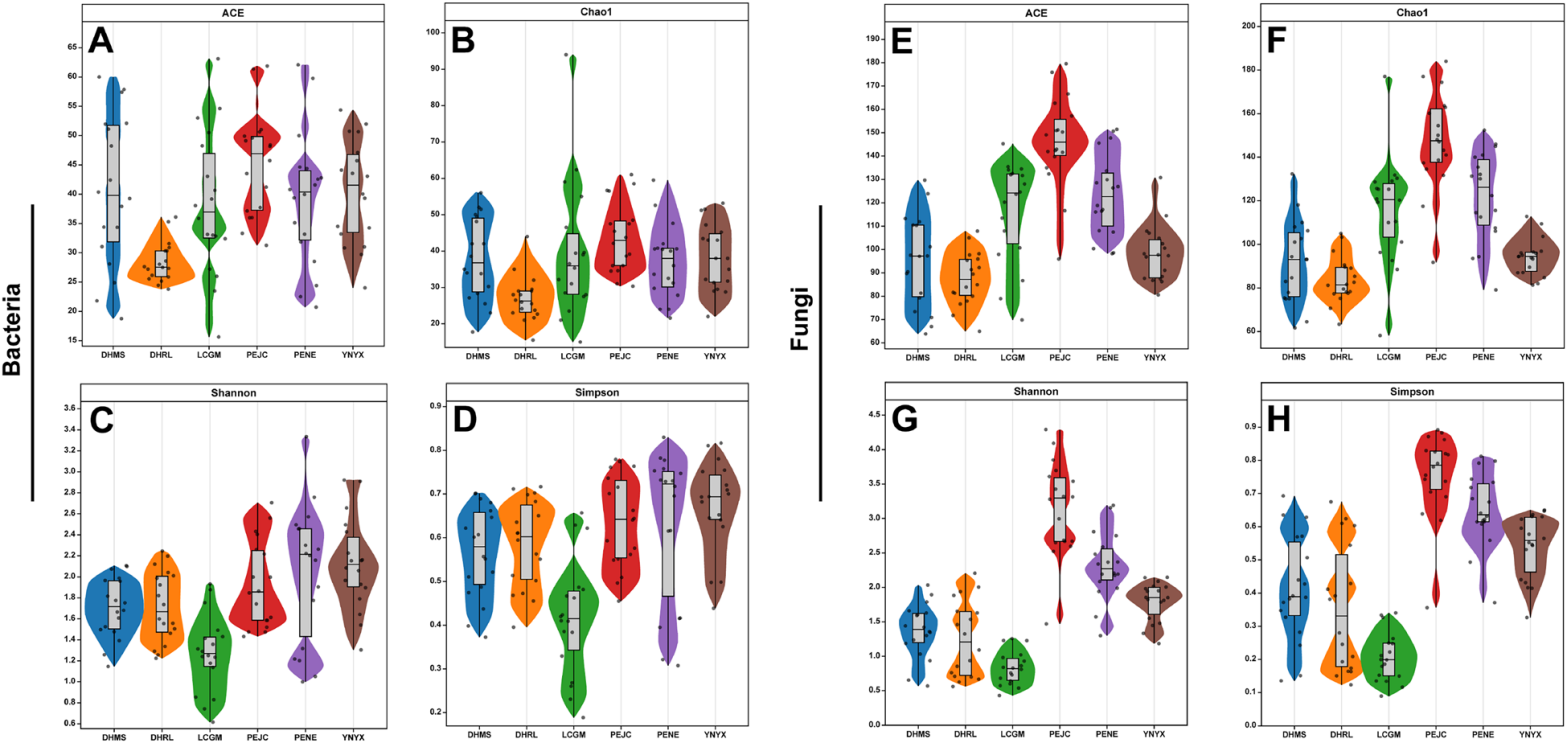
The alpha diversity of microbial communities in different regions filler tobacco leaves (see Additional file 1: Table S4). Alpha diversity indices of bacteria, **A** Ace indices, **B** Chao1 richness, **C** Shannon indices and **D** Simpson indices; Alpha diversity indices of fungi, **E** Ace indices, **F** Chao1 richness, **G** Shannon indices and **H** Simpson indices

Alpha diversity indices, including Ace, Chao 1, Shannon, and Simpson, were employed to assess microbial community richness and diversity. As depicted in Fig. 3A, B, DHRL exhibited significantly lower Ace and Chao1 indices for bacterial communities in comparison to other groups. In Fig. 3C, D, it is apparent that the Shannon and Simpson indices of bacterial communities in LCGM were notably lower than those in other groups (*p* < 0.05, as detailed in Additional file 1: Table S9). This suggests that while variations in bacterial community abundance and diversity exist, the overall pattern remains relatively consistent following integrated FTLs fermentation. This further indicates that bacterial community structure exhibits a degree of stability and homogeneity, with less pronounced influence from geographical factors. Shifting our focus to fungal communities (Fig. 3E, F, G and H), it is evident that PEJC displayed higher Ace, Chao, Shannon, and Simpson indices in comparison to the other groups (*p* < 0.05, as detailed in Additional file 1: Table S9). The variations in abundance and diversity among fungal communities across the six distinct FTLs samples highlight that, unlike bacteria, the microbial community structure of fungi is considerably more susceptible to geographic influences.

**Fig. 4:**
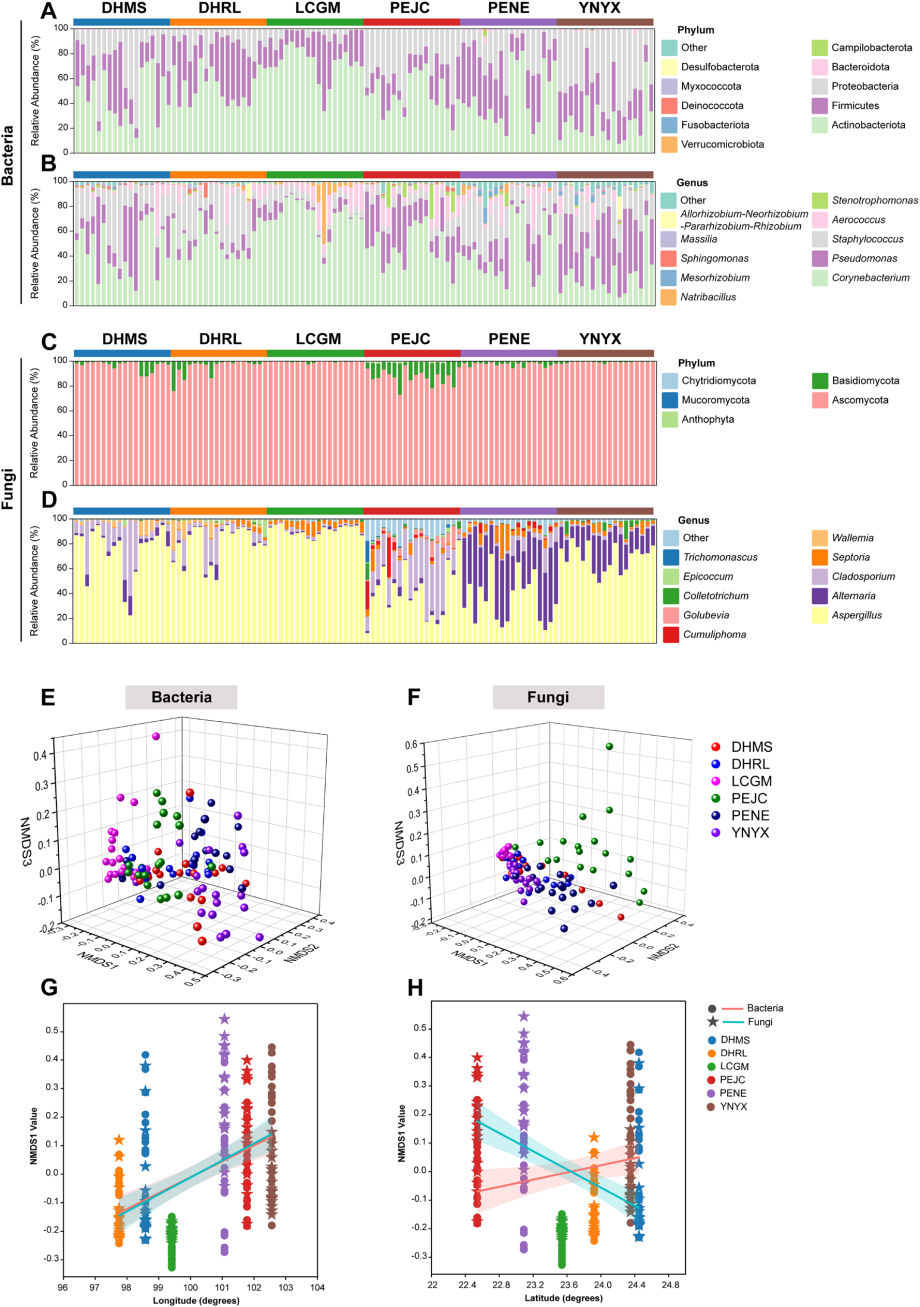
Microbial characteristics of DHMS, DHRL, LCGM, PEJC, PENE and YNYX in 108 samples. Phylum (**A**) and genus (**B**) that were detected at a rank of top 10 in this survey for bacterial and phylum (**C**) and genus (**D**) of fungi. Nonmetric multidimensional scaling (NMDS) plot based on the structures of the bacterial (**E**) and fungal (**F**) communities. Microbial NMDS1 is significantly linked to geographic longitude (**G**) and latitude (**H**). The lines represent the regression line fitted by the first-order polynomial

The microbial community composition structures displayed significant heterogeneity among FTLs samples originating from diverse geographical regions. However, following integrated fermentation, *Actinobacteriota*, *Firmicutes*, and *Proteobacteria* emerged as the predominant bacterial phyla across all samples (Fig. 4A). *Actinobacteriota* exhibited a mean relative abundance of 47.88%, with variability ranging from 72.37% to 25.37% among different geographical areas. Notably, LCGM samples displayed the highest relative abundance of *Actinobacteriota* (72.37%), while YNYX samples had the lowest (25.37%). Intermediate values were observed in DHMS, DHRL, PEJC, and PENE samples, ranging from 44.46% to 53.34%. Total 103 bacterial genera were found in all samples, at the genus level, with *Corynebacterium* (47.68%), *Pseudomonas* (19.95%), *Staphylococcus* (18.29%), and *Aerococcus* (7.19%) being the predominant genera. The relative abundance of *Corynebacterium* and *Pseudomonas* exhibited variations within the range of 24.92% to 72.32% and 4.37% to 39.46%, respectively, during fermentation. The relative abundance of *Staphylococcus* and *Aerococcus* in samples from different geographic regions ranged from 7.20% to 23.49% and 4.83% to 9.75%, respectively (Fig. 4B). In the fungal communities, the dominant phylum was *Ascomycota*, prevalent in samples from various geographical regions (Fig. 4C). A total of 132 genera at the genus level were recognized, with YNYX samples containing only 90 genera, whereas samples from other geographic regions comprised over 100 genera. The most predominant genera were *Aspergillus* (65.10%), *Alternaria* (12.75%), and *Cladosporium* (10.69%). The relative abundance of *Aspergillus*, *Alternaria*, and *Cladosporium* exhibited variations ranging from 35.03% to 90.91%, 0.90% to 45.13%, and 1.50% to 28.79%, respectively, across samples from different geographic regions. *Aspergillus* dominated the LCGM samples with a relative abundance of 90.91% and ranged from 35.03% to 79.67% in other groups. Notably, the relative abundance of *Alternaria* varied significantly among different geographic regions, accounting for 5.04%, 2.16%, 0.90%, 2.65%, 45.13%, and 20.64%, respectively. Regarding *Cladosporium*, it exhibited a high relative abundance in PEJC samples (28.79%) and a low relative abundance in DHRL samples (1.50%), with intermediate values ranging from 2.32% to 16.15% among DHMS, DHRL, PEJC, and PENE samples (Fig. 4D).

Our analysis revealed that the most abundant microbial genera, with a relative abundance exceeding 1%, were consistent across geography-dependent aroma groups, collectively constituting over 90% of the total relative abundance for both bacteria and fungi (Fig. 4). Here is a summary of these predominant genera for each group. For DHMS samples, the most abundant bacteria were *Corynebacterium*, *Pseudomonas*, *Staphylococcus*, and *Aerococcus*. Among fungi, *Aspergillus*, *Alternaria*, *Cladosporium*, and *Wallemia* were the dominant genera. In DHRL samples, *Corynebacterium*, *Pseudomonas*, *Staphylococcus*, *Aerococcus*, and *Sphingomonas* were the most prevalent bacteria, while the primary fungi included *Aspergillus*, *Alternaria*, *Cladosporium*, *Septoria*, *Wallemia*, and *Epicoccum*. Within LCGM samples, *Corynebacterium*, *Pseudomonas*, *Staphylococcus*, *Aerococcus*, and *Natribacillus* were the predominant bacteria, while *Aspergillus*, *Cladosporium*, and *Septoria* were the dominant fungi. PEJC samples exhibited *Corynebacterium*, *Pseudomonas*, *Staphylococcus*, *Aerococcus*, and *Stenotrophomonas* as the major bacteria, while the primary fungi encompassed *Aspergillus*, *Alternaria*, *Cladosporium*, *Septoria*, *Cumuliphoma*, *Golubevia*, *Colletotrichum*, and *Trichomonascus*. In the case of PENE samples, the most abundant bacteria included *Corynebacterium*, *Pseudomonas*, *Staphylococcus*, *Aerococcus*, *Stenotrophomonas*, *Natribacillus*, and *Mesorhizobium*, whereas the dominant fungi were *Aspergillus*, *Alternaria*, *Cladosporium*, *Septoria*, and *Cumuliphoma*. For YNYX samples, the major bacteria comprised *Corynebacterium*, *Pseudomonas*, *Staphylococcus*, *Aerococcus*, *Mesorhizobium*, *Massilia*, and *Allorhizobium-Neorhizobium-Pararhizobium-Rhizobium*. Among fungi, *Aspergillus*, *Alternaria*, *Cladosporium*, *Septoria*, and *Colletotrichum* were the dominant genera.

Interestingly, the dominant genera were consistent across all six groups, albeit with varying relative abundances. PENE and PEJC exhibited the highest number of unique bacterial and fungal genera within fermented FTLs (Additional file 1: Fig. S5). Statistically, the microbial structures at the genus taxonomic level among the six groups showed significant differences (*p* < 0.05) (Fig. 4E, F). Regarding fungal structures, the PEJC and PENE groups were notably separated from the DHMS, DHRL, LCGM, and YNYX groups along the nonmetric multidimensional scaling 1 (NMDS1). Furthermore, the PEJC and PENE groups exhibited separation along NMDS3. The stress value of 0.05 in fungal community differentiation among the six groups signifies the robustness and reliability of the NMDS analysis results. The six groups' division into bacterial genera could not be distinguished as clearly as that seen in fungi, suggesting that the differences in fungal structures may be more pronounced. Both fungal and bacterial structures displayed significant correlations with latitude and longitude (Fig. 4G, H). The fungal structure displayed significant linear correlations with both longitude (R2 = 0.229; *p* < 0.001) and latitude (R2 = 0.251; *p* < 0.001), emphasizing the geographical dependence of fermentation fungal genera (Fig. 4G, H and Additional file 1: Fig. S6). However, the bacterial structure exhibited a weaker correlation with longitude (R2 = 0.206; *p* < 0.001), and the correlation with latitude was insignificant. This implies that fermentation bacterial genera are less influenced by geographical factors compared to fungal genera.

### Correlation of the microbial community with chemical constituents and volatile organic compounds

**Fig. 5:**
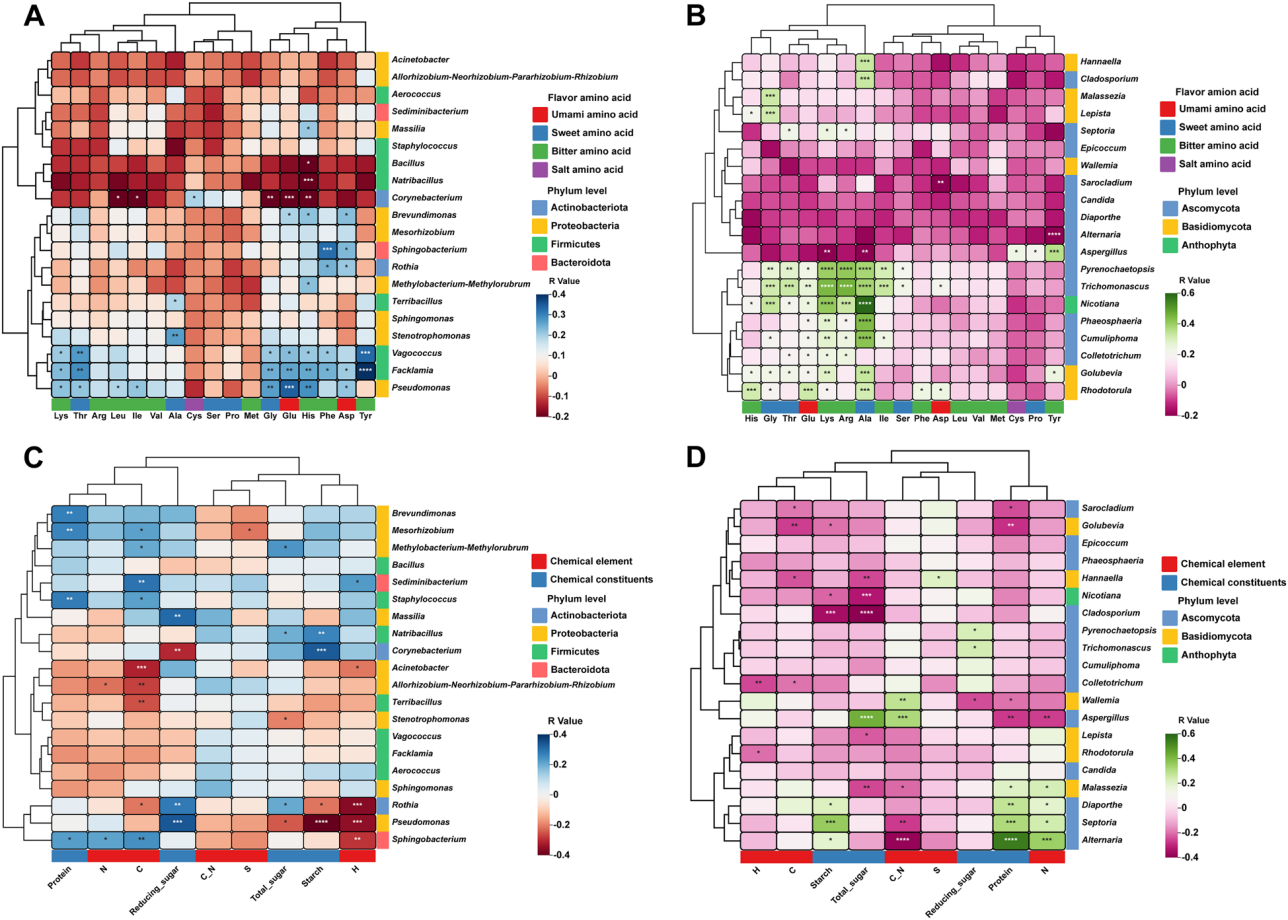
Correlation analysis between microbial flora (relative abundance of the top 20) with FAAs, chemical elements, and chemical constituents of filler tobacco leaves. Heat map of correlation between bacterial (**A**) with fungal (**B**) and FAAs; heat map of correlation between bacterial (**C**) with fungal (**D**) and chemical elements and chemical constituents; Blue and green represent positive correlation, red and purple represent negative correlation. Significant correlations were expressed by *, * *,* * * and * * * * which were *p* < 0.05, *p* < 0.01, *p* < 0.001 and *p* < 0.0001, respectively

In this study, we identified 24 marker VOCs, 17 FAAs, chemical constituents, and chemical elements within the six groups of FTLs. To explore the relationship between microbial communities and these chemical constituents and FAAs, we evaluated the relative abundance of the top 20 microbial genera (bacteria and fungi) at the genus level. We determined the associations between these biomarkers and the indexes of chemical constituents and FAAs using Pearson correlation analysis. Figure 5A, B illustrates that the biomarkers displayed both positive and negative correlations with FAAs, while Fig. 5C, D reveals that these biomarkers exhibited both positive and negative correlations with chemical constituents and chemical elements. Among the FAAs significantly correlated with bacterial microorganisms, the primary contributors included Lys, Thr, Ala, Gly, Glu, His, Phe, Asp, and Tyr. Conversely, among the FAAs significantly correlated with fungal microorganisms, the key contributors were His, Gly, Thr, Glu, Lys, Arg, and Ala. These FAAs primarily belong to the categories of sweet and bitter amino acids. Bacterial microorganisms that exhibited significant positive correlations with FAAs were primarily represented by *Vagococcus*, *Facklamia*, and *Pseudomonas*, all within the *Firmicutes* and *Proteobacteria* phyla. Notably, only *Pseudomonas* displayed a significant correlation with reducing sugar, total sugar, starch, and the element H. Conversely, fungal microorganisms that displayed significant positive correlations with FAAs included *Pyrenochaetopsis*, *Trichomonascus*, *Phaeosphaeria*, *Cumuliphoma*, *Colletotrichum*, *Golubevia*, *Rhodotorula*, and *Nicotiana*, representing the *Ascomycota*, *Basidiomycota*, and *Anthophyta* phyla. In contrast, microorganisms within the *Ascomycota* and *Basidiomycota* genera, such as *Malassezia*, *Diaporthe*, *Septoria*, *Alternaria*, *Wallemia*, and *Aspergillus*, demonstrated more significant interactions with chemical elements and chemical constituents. This suggests that fungal microorganisms may have a broader influence on metabolic products.

**Fig. 6:**
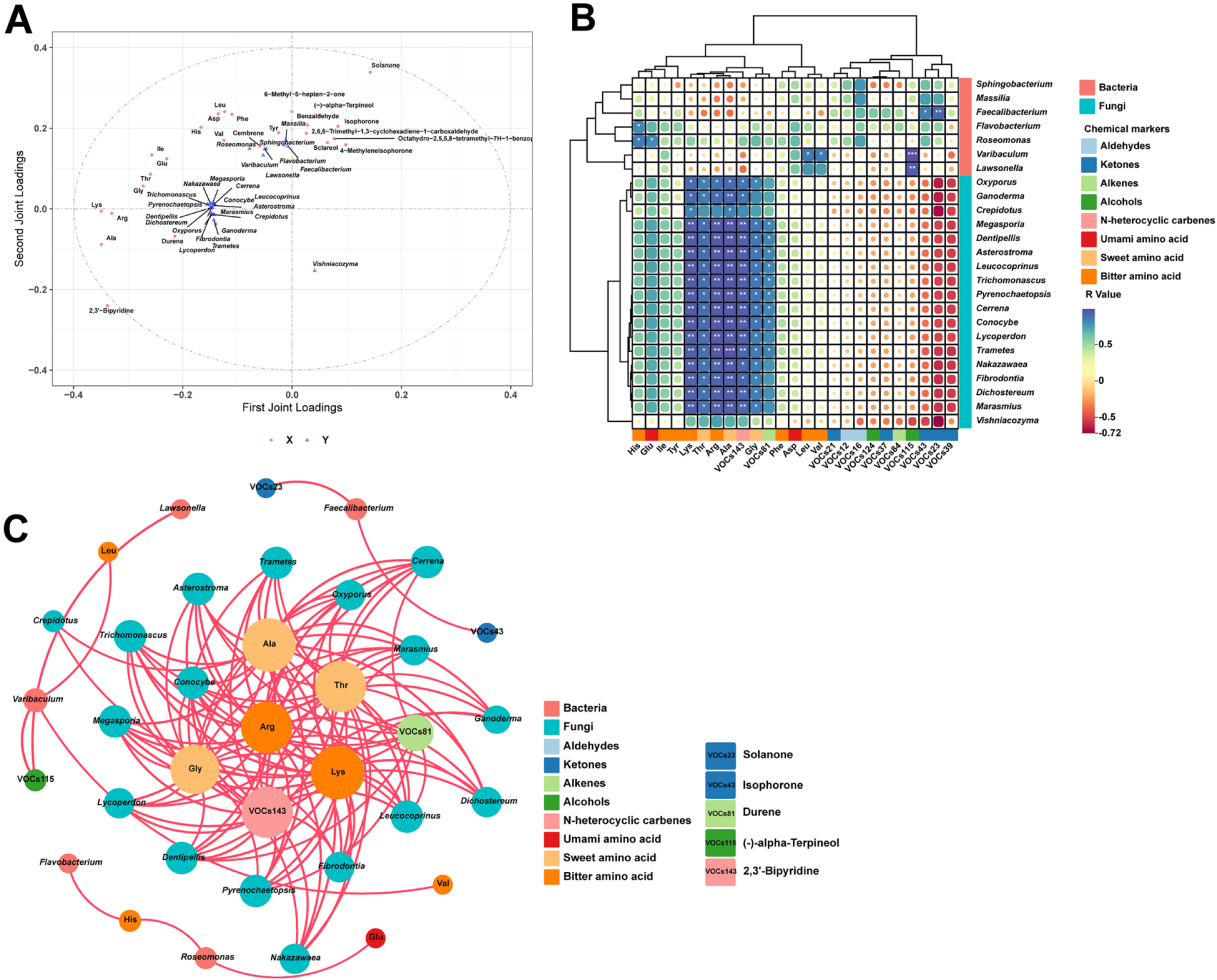
Correlation analysis between microbial flora and metabolites of filler tobacco leaves. **A** Load map of microbial flora and metabolite prediction model based on O2PLS; **B** Heat map of correlation between microbial flora with VOCs and FAAs (VOCs and FAAs were screened based on O2PLS), blue represents positive correlation, red represents negative correlation; Significant correlations were expressed by *, * *, and * * *, which were *p* < 0.05, *p* < 0.01 and *p* < 0.001, respectively. **C** Pearson’s correlation coefficient (|*r*|> 0.7, *p* < 0.05) indicates robust correlations. The different color circles refer to bacteria, fungi, volatile organic compounds, and free amino acids, respectively. VOCs23, VOCs43, VOCs81, VOCs115 and VOCs143 denote solanone, isophorone, durene, (-)-alpha-terpineol, and 2,3'-bipyridine, respectively. The red lines refer to positive (*r* > 0.7) correlations. The size of nodes indicates the degree of connections

We conducted an extensive analysis to elucidate the intricate relationships among microbial genera, VOCs characterized by VIP scores exceeding 1.0, FAAs, and chemical elements within the context of FTLs. This comprehensive investigation involved the utilization of the O2PLS model and the Pearson correlation coefficient, as illustrated in Fig. 6A. The R2 values for the metabolome and microbiome were 0.607 and 0.458, respectively, indicating that the O2PLS model had good interpretative and predictive capabilities. In preparation for the O2PLS analysis, we selected microbial genera with a relative abundance exceeding 0.1%. This data curation process led to the inclusion of 25 microbial genera, comprising 7 bacterial and 18 fungal genera. Additionally, our analysis encompassed 12 VOCs and 13 FAAs, representing a diverse array of compounds, including 2 aldehydes, 5 ketones, 2 alkenes, 2 alcohols, 1 n-heterocyclic carbene, 2 umami amino acids, 3 sweet amino acids, and 8 bitter amino acids.

Based on the modeling results using O2PLS, a deeper study of the correlation between microbial genera and metabolites was conducted using the correlation coefficient matrix. It was determined that VOCs and FAAs were closely associated with the network model of microbes. As shown in Fig. 6B, bacterial microorganisms and fungal microorganisms formed distinct clusters. Notably, fungal microorganisms had a more significant impact on metabolites. This could be attributed to the fact that fungal microbial community structures are more influenced by geographical factors, and their region-specific microbial communities have a greater impact on metabolite production. In a previous study, it was found that the microbiomes of baijiu, along with their metabolites, were associated with flavor and influenced by geographical factors (Tan et al. 2022). Using Pearson correlation coefficients, the relationship between microbial communities and VOCs and FAAs (selected through O2PLS) was investigated. A total of 625 pairwise correlations were analyzed, and 115 strong correlations were identified based on criteria including |*r*|> 0.7 and p < 0.05. These correlations were visualized using Gephi (Fig. 6C), and all of them were positive (indicated by red curves). Among the bacterial genera, *Varibaculum* showed a positive correlation with Leu, Val, and (-)-alpha-terpineol. *Faecalibacterium* exhibited a positive correlation with solanone and isophorone. *Roseomonas* had a positive correlation with His and Glu. *Flavobacterium* and *Lawsonella* were positively correlated with His and (-)-alpha-terpineol, respectively. Within the fungal genera, *Oxyporus*, *Megasporia*, *Dentipellis*, *Asterostroma*, *Leucocoprinus*, *Trichomonascus*, *Pyrenochaetopsis*, *Cerrena*, *Conocybe*, *Lycoperdon*, *Trametes*, *Nakazawaea*, *Fibrodontia*, *Dichostereum*, and *Marasmius* were positively correlated with Lys, Thr, Arg, Ala, Gly, and 2,3'-bipyridine. Additionally, *Megasporia*, *Dentipellis*, *Asterostroma*, *Leucocoprinus*, *Trichomonascus*, *Pyrenochaetopsis*, *Cerrena*, *Conocybe*, and *Trametes* were also positively correlated with Durene. This analysis indicates that the FAAs and VOCs in different regions of FTLs are significantly influenced by fungi.

## Discussion

Tobacco leaf quality typically encompasses external appearance, physical characteristics, and intrinsic quality, with intrinsic quality primarily referring to the harmony of various chemical components and their proportions within the tobacco leaves (Tso 1972). Additionally, unlike the curing process of cigarette tobacco leaves, cigar tobacco undergoes a lengthy process of air-curing, fermentation, and aging, resulting in distinct classification and attribute characteristics compared to cigarette tobacco (Yan et al. 2021). In the results of this study, the overall chemical composition of tobacco leaves in different regions is relatively harmonious. However, there were variations in some chemical components and aroma compounds among different cigar tobacco samples. Studying these differences in compounds of cigar tobacco leaves from different cultivation regions is crucial, as it provides valuable insights for implementing tailored fermentation processes and targeted improvements in the future. These findings suggest that compounds such as pyridine, 3-(1-methyl-2-pyrrolidinyl)-, (S)-pyridine, 3-(3,4-dihydro-2H-pyrrol-5-yl)-, and nicotyrine derived from n-heterocyclic carbenes play a pivotal role in shaping the flavor profile of cigar tobacco leaves. Nitrogen compounds and carbohydrate substances represent pivotal conventional chemical constituents in tobacco leaves, with their content bearing substantial implications for the overall tobacco quality. Notably, the reactions of carbohydrate substances and nitrogen compounds during tobacco leaf combustion exert divergent effects on flavor. Carbohydrate substances' pyrolysis products yield an acidic reaction, while nitrogen compounds, particularly alkaloids, generate a basic reaction. It is the harmonious balance of these two components that underpins the creation of a favorable flavor profile (Su et al. 2017).

Findings from this investigation underscore a distinctive characteristic of cigar tobacco: its relatively low carbohydrate substance content. This is a consequence of the prolonged and consistent air-curing and fermentation processes that cigar tobacco undergoes. Consequently, there are minimal disparities in the content of umami amino acids, sweet amino acids, and cysteine among various cigar tobacco samples. However, when juxtaposed with cigarette tobacco, cigar tobacco still manifests notable distinctions in amino acid type distribution (Zhao 2012). Examining the average content of diverse identified constituents, it becomes evident that the LCGM region exhibits a significantly lower mean content of reducing sugars in comparison to the YNYX region. Furthermore, the DHMS, PEJC, and PENE regions display a notably lower mean total sugar content relative to the DHRL, LCGM, and YNYX regions. Additionally, the PENE region registers a significantly higher starch content compared to the DHRL and PEJC regions. Noteworthy differences in starch content are also discernible among cigar tobacco samples sourced from distinct regions. While tobacco quality is undeniably linked to the content and proportion of its chemical constituents, it is essential to acknowledge that it cannot be solely determined by the quantity of these constituents. Nevertheless, the observed distinctions in carbohydrate content, chemical element composition, amino acids, and other constituent levels across tobacco samples from diverse regions emphasize the continued significance of advancing the homogenization of tobacco leaves.

Transitioning from the chemical composition to the aromatic profile, tobacco aroma serves as a fundamental criterion for assessing the inherent quality of tobacco leaves. The quantity, character, and variety of tobacco aroma are contingent upon the composition, quantity, ratio, and interplay of numerous aromatic constituents. These constituents, each with its own distinct concentration, bestow upon cigar tobacco its distinctive olfactory attributes, setting it apart from other types of tobacco (Tso 1972). In the course of this investigation, 24 VOCs were identified as pivotal volatile elements contributing to the variances in flavor profiles discernible among cigar tobacco leaves sourced from six distinct cultivation regions. Employing O2PLS modeling analysis, five VOCs, namely solanone, isophorone, durene, (-)-alpha-terpineol and 2,3'-bipyridine were singled out as the VOCs exhibiting the most pronounced correlation with microbiome data. Solanone, a crucial flavoring agent in tobacco, originates from the biochemical degradation of sesquiterpenes inherent to tobacco. As tobacco undergoes processing and aging, solanone undergoes further transformations, giving rise to compounds like somnirol, solanofuran, and norsolandione. These derivatives exert a considerable influence on the flavor profiles of cigarettes (Johnson and Nicholson 1965). Conversely, isophorone is a degradation byproduct of carotenoids and belongs to the terpenoid family. This aromatic constituent is encountered in various tobacco varieties and is celebrated for its potent and enduring fragrance, characterized by subtle undertones of slight acidity, honeyed sweetness, woody notes, and dried fruit essences. It significantly enhances the flavor profiles of diverse tobacco types (Zhou et al. 1997). Durene, an aromatic compound, is primarily prevalent in tobacco smoke. As for (-)-alpha-terpineol, it emanates a fragrance reminiscent of lilac and occurs naturally in several plants. Classified as a monoterpene, (-)-alpha-terpineol boasts a diverse range of pharmacological properties, including antioxidant, antibacterial, antifungal, anticancer, antiulcer, and potent anti-inflammatory attributes (Khan et al. 2023). Additionally, 2,3'-bipyridine, also recognized as nornicotine or isonicotine, represents a heterocyclic organic compound pivotal within the class of nitrogen-containing compounds found ubiquitously in tobacco and tobacco products like nicotiana tabacum (Chen et al. 2010). It is of note that solanone and isophorone exhibited notably higher mean concentrations in the YNYX region relative to other regions. This suggests that cigar tobacco samples sourced from the YNYX region may possess a more propitious foundation concerning crucial aroma components.

The withering and fermentation phases exert decisive influence over the creation of aroma compounds in cigar tobacco leaves. Fermentation, in particular, is widely recognized as a harmonious interplay of chemical reactions, microbial activity, and enzyme-mediated processes (Wang 2003). Microorganisms, in particular, are deemed pivotal contributors to the enhancement of cigar tobacco leaf quality (Gribbins et al. 1944). In this study, we probed the structural and diversity aspects of microbial communities within FTLs originating from various agricultural regions. To accomplish this, Illumina MiSeq sequencing, targeting the 16S rRNA and ITS genes, was employed. The outcomes illuminated significant disparities in fungal communities across FTLs derived from distinct locales. *Actinobacteria*, *Firmicutes*, and *Proteobacteria* were most prevalent at the phylum level in the FTLs, which is in line with findings from earlier studies on flue-cured tobacco leaves (Zhang et al. 2020a). In our analysis of fermented FTLs from Yunnan, we observed a pronounced prevalence of the fungal phylum *Ascomycota* and the bacterial phylum *Actinobacteriota*. These findings are in concordance with the microbial patterns reported in H382 cigar leaves from Hainan (Liu et al. 2021a). Further corroborating our results, Zhang et al. (Zhang et al. 2023a) documented the dynamic interplay of microbial communities during leaf stacking fermentation, highlighting their critical role in the transformation of macromolecules and the synthesis of distinctive volatile aroma compounds. The study also revealed that both the fermentation stage and geographic location influence microbial diversity, particularly impacting core taxa like *Sphingomonas*, *Bacillus*, *Aspergillus*, and *Penicillium*. These comprehensive analyses collectively enhance our understanding of how regional microbial communities contribute to the unique flavor profiles and chemical characteristics of cigar tobacco leaves, affirming the integral role of microbiota in the fermentation process and its influence on the quality of tobacco products. At the genus level, *Corynebacterium*, *Pseudomonas*, *Staphylococcus*, *Aerococcus*, *Aspergillus*, *Alternaria*, and *Cladosporium* were the leading genera. These taxa were also dominant in the FTLs that occurred in Mexico (Zhang et al. 2018) and Hainan (Zhang et al. 2021). Research by Zheng et al. (Zheng et al. 2022) underscored the pivotal contributions of *Corynebacterium* and *Staphylococcus* to flavor formation in cigar tobacco leaves. Additionally, the salt-tolerant and resistant *Aerococcus*, renowned for these traits, has been reported as a dominant microorganism in the curing process of jatropha shoots. This resilient microbe has also been detected in studies involving FTLs, where it harnesses the reducing sugars, malic acid, and citric acid present in tobacco leaves to elevate temperature and pH levels. This, in turn, facilitates the proliferation of salt-tolerant and resistant *Corynebacterium* (Li et al. 2020). *Alternaria* demonstrates its prowess in breaking down cellulose within FTLs (Macris 1984). As previously highlighted, *Cladosporium* emerged as the predominant fungal genus, confirming our research findings (Zhang et al. 2023b; Hu et al. 2022). Post-fermentation, the dominant microbes observed were *Pseudomonas*, *Staphylococcus*, and *Aspergillus*. These microorganisms play pivotal roles across various processes, encompassing the degradation of nicotine, (Li et al. 2010) pectin, protein, starch, (Barbesgaard et al. 1992) xyloside, and oligosaccharides (Coenye 2014). Moreover, they contribute to the generation of fatty acids and amino acids, (Tabanelli et al. 2012) precipitating the development of an array of flavor compounds and carbohydrates, ultimately elevating the quality of FTLs. However, it's worth noting that while *Aspergillus* may enhance cigar leaf quality by reducing nicotine levels or generating organic acids like citric acid, it may also be associated with mildew issues in cigar leaves. Furthermore, research has suggested that the microbial communities within fermenting cigar tobacco leaves do not solely originate from the soil in which the tobacco plants are cultivated. Instead, during the fermentation process, these communities are gradually recruited from the surrounding environment (Zhang et al. 2023c). The transformations in community structure and composition are primarily correlated with shifts in FTLs, arising from changes in their metabolic functions (Zhou et al. 2020). Microbes play a significant role in synthesizing amino acids, fatty acids, and metabolizing aromatic compounds, which ultimately contribute to the enhancement of the flavor and aroma of FTLs (Wang 2003).

In the results of this study, the average levels of conventional chemical components in cigar tobacco samples from different regions of Yunnan generally fell within the appropriate ranges for each component, indicating a relative harmony in their chemical composition. Further, our findings highlight the metabolic activities of microorganisms during the fermentation of cigar tobacco leaves. The microbial-mediated hydrolysis of peptides and/or proteins, coupled with the progression of the Maillard reaction, contributes significantly to alterations in the distribution of FAAs, shaping the different flavor profile of cigar tobacco leaves (Tao et al. 2014). Additionally, our application of O2PLS modeling analysis to examine the correlation between microbial genera and metabolites. This method has allowed us to identify specific VOCs that exhibit a strong correlation with microbiome data, enhancing our understanding of the microbial contribution to tobacco flavor. Specifically, seven bacterial genera and 18 fungal genera were identified as having the highest associations with metabolites. Notably, the impact of fungal microorganisms on metabolites was more pronounced, which has a stronger regional impact on microbial communities than bacteria, with the dominant fungal phyla mainly including *Ascomycota* and *Basidiomycota*. *Ascomycota* is a common endophytic fungal group found in plants (Angelini et al. 2012). Chen et al. (2018) reported that *Ascomycota* is a dominant fungal phylum shared among tobacco leaves from different regions and parts. It has the ability to influence amylase activity. *Basidiomycota*, on the other hand, is a dominant fungal phylum in the later stages of cigar tobacco leaf fermentation. The predominant phyla identified in our study align with findings from numerous other studies related to the tobacco leaf microbiome. These phyla are known to take part in the assimilation of starch, xylan, and cellulose, as well as other carbon degradation processes. They perform the role of decomposers by converting complex molecules like cellulose, pectin, and starch into less complex ones like glucose, fructose, and maltose during the fermentation of tobacco (Costa et al. 2020). While our study provides valuable insights, it also presents some limitations. The scope of microbial diversity analysis could be expanded in future research to encompass a broader array of microbial taxa and their specific roles in tobacco fermentation. The generalization of our findings may be limited to the specific regions and tobacco types studied, highlighting the need for future studies to broaden these findings' applicability. Additionally, our focus on dominant microbial genera might have overshadowed the contributions of rare microorganisms in the fermentation process.

## Conclusion

In conclusion, our study underscores the significant role of environment and processing in shaping the chemical composition and thereby the quality and style of tobacco leaves. The ecological conditions of the growing region emerge as a crucial factor influencing the intrinsic characteristics of the tobacco leaves. Despite uniform fermentation across various Yunnan regions, there is still room for improvement in the homogeneous development of FTL samples, suggesting the possibility of unified regional development through tailored agricultural fermentation strategies. Notably, our results reveal that fungi, compared to bacteria, exhibit stronger regional characteristics in shaping the microbial community structure of different cultivation areas. These findings may aid in guiding the optimization of cultivation and fermentation practices, enhancing tobacco leaf quality and potentially benefiting tobacco farmers economically.

### Supplementary Information

**Additional file 1: Figure S1.** FTLs sampling diagram, DHMS, DHRL, LCGM, PEJC, PENE, and YNYX respectively denote distinct geographical region names. **Figure S2.** Principal component analysis (PCA) based on chemical elements and chemical constituents of six regions of FTLs. **Figure S3.** The Linear Discriminant Analysis (LDA) plots of sensory evaluation of the six groups of FTLs. **Figure S4.** (A) The heatmap of volatile chemicals in the six groups fermented FTLs identified via HS–SPME–GC–MS detection. (B) Relative concentration of chemicals in the end of fermentation. (C) and (D) The Venn diagram of volatile chemicals in the six groups fermented FTLs. **Figure S5.** Venn plot of bacterial and fungal diversity at genera taxonomic level. **Figure S6.** The microbial NMDS2 (A, B) and NMDS3 (C, D) were linked to the geography longitude (A, C) and latitude (B, D). The lines represent the regression line fitted by the first-order polynomial. **Table S1.** Information of filler tobacco leaves (FTLs). **Table S2.** Analysis of free amino acids of the six groups FTLs. **Table S3.** Analysis of different species free amino acids in FTLs. **Table S4.** Analysis of chemical elements concentrations of the six groups FTLs. **Table S5.** Analysis of chemical constituents of the six groups FTLs. **Table S6.** Relative concentration of volatile chemicals in the six groups fermented FTLs identified via HS–SPME–GC–MS detection. **Table S7.** VIP values of volatile chemicals based on partial least squares-discrimination analysis model among six groups. **Table S8.** The numbers of microbes at phyla, class, family, genus and OTU level in six group fermented FTLs. **Table S9.** Alpha diversity indices of the microbial community in FTLs samples.

## Data Availability

Data and materials described in this study are available from the authors upon reasonable request and availability.
